# Interaction of semen with female reproductive tract tissues: what we know, what we guess and what we need to do

**DOI:** 10.1590/1984-3143-AR2024-0042

**Published:** 2024-08-05

**Authors:** John James Bromfield

**Affiliations:** 1 Department of Animal Sciences, University of Florida, Gainesville, FL, USA

**Keywords:** postcoital inflammation, semen, seminal fluid

## Abstract

For nearly 100 years the postcoital inflammatory response has been described in the female reproductive tract of rodents. Since the 1950’s this observation has been made in a number of animals including humans and domestic species. Yet pregnancy can be initiated and maintained by using embryo transfer which bypasses insemination and the related postcoital inflammatory response. Thus, the role of semen exposure beyond sperm transport and subsequent postcoital inflammatory response in female reproductive tissues has yet to be given a true physiological purpose. Historically the postcoital inflammatory response of female tissues was suggested to remove spermatozoa and male derived pathogens from the female reproductive tract. More recently, semen exposure and the postcoital inflammatory response have been suggested to play a role in long-term preparation of the maternal immune system to the semi-allogeneic pregnancy, ancillary support of the preimplantation embryo, and potentially fetal programing that improves pregnancy outcomes, while the absence or inappropriate postcoital inflammation has been suggested to contribute to pregnancy complications. Although the postcoital inflammatory response has been robustly characterized, the evidence for its role in promoting positive pregnancy outcomes or reducing pregnancy complications remains tenuous. This manuscript is designed to balance the information we know regarding semen exposure and postcoital inflammation in various animal systems, with the information we perceive to be factual but perhaps not yet fully tested, along with the data we have yet to generate if we intend to postulate a physiological purpose of the postcoital inflammatory response to pregnancy outcomes.

## Introduction

Semen is a complex biological fluid comprised of spermatozoa, paternal somatic cells, bacteria, and acellular seminal fluid that contains numerous bioactive molecules, substrates, salts, minerals, and various other compounds. The main purpose of semen is to deliver spermatozoa through the male reproductive tract and into the female reproductive tract while supporting the viability of spermatozoa required for fertilization. While the interactions between semen and female reproductive tract tissues have been extensively studied in rodent models, details of a similar response in other animals are lacking (Bromfield, 2018). In this light, it is important to highlight the significant difference in how and where semen is deposited into the female reproductive tract of various species. In rodents the entire uterus is filled with semen at the time of ejaculation, similar to the intracervical insemination that occurs in swine; however, in most other species semen is deposited into the vagina and only a small fraction of spermatozoa traverse the cervix whilst the majority of semen is lost through retrograde transport from the vagina (Marey et al., 2023). It is therefore unclear if the type or degree of postcoital inflammation or secondary responses observed in rodents is similar to that of other species and what the consequence of these responses is to pregnancy. This review will focus on what is known regarding the interactions of semen with female tissues and what requires enlightenment if semen has utility in pregnancy outcomes beyond facilitating fertilization in relevant species for agriculture or human health.

## What we know about how semen interacts with tissues of the female reproductive tract

In 1929 Donald Yochem performed a series of detailed experiments to evaluate the survival of spermatozoa in the female reproductive tract of the guinea pig after copulation or artificial insemination (Yochem, 1929). Collectively his work demonstrated a rapid clearance of spermatozoa from the uterus after copulation and he posited that “I have been unable to satisfy myself as to the exact method of their [sperm] elimination, but believe it to be largely due to the phagocytic action of cells within the lumen of the tract.” Since then, a series of experiments have further characterized the inflammatory response of female reproductive tissues to semen, including an elegant series of experiment performed by the reproductive biology pioneers, Ryuzo Yanagimachi and M. C. Chang who describe that postcoital inflammation in hamsters is halved if epididymal sperm were injected into the uterus compared to mating with intact or vasectomized males (Yanagimachi and Chang, 1963). These men also tested the capacity of mechanical stimulation of the female reproductive tract to induce inflammation and concluded that postcoital inflammation was only induced if females were exposed to an intact male or seminal fluid (Table 1). At this time, it was queried by the authors as to whether there were other functions of postcoital inflammation other than clearance of spermatozoa. Secondary to these observation in various rodent species, the acute postcoital inflammatory response to semen in the first 24 hours after insemination has been characterized in human, cattle, swine, horse and goats (Mattner, 1968; Fumuso et al., 2003; O'Leary et al., 2004; Sharkey et al., 2007; Marey et al., 2023).

**Table 1:** Number of uterine leucocytes recovered from the hamster after mating.

**Treatment applied to female (*n*)**	**Mean number of uterine leukocytes recovered after 12-14 h (range)**
Mated to intact male (*7*)	927,300 (61,000 – 3,580,000)
Mated to vasectomized male (*8*)	662,100 (32,000 – 2,244,000)
Epididymal sperm injected into the uterus (*7*)	436,000 (18,000 – 2,220,000)
Seminal fluid injected into the uterus (*7*)	273,000 (9,000 – 990,000)
Injection of Hanks solution into the uterus (*7*)	79,500 (7,000 – 258,000)
Ligation of the cervix (*7*)	47,000 (1,000 – 216,000)
Ligation of the cervix and mated to intact male (*5*)	12,500 (1,000 – 250,000)

This table was adapted from the foundational work of Yanagimachi and Chang (1963).

It wasn’t until the 1990’s that the interest in postcoital inflammation was reenergized, and a physiological role for insemination beyond delivery of spermatozoa was investigated. Comprehensive studies in the rodent demonstrate numerous effects in maternal tissues after semen exposure. Initially the same acute, transient postcoital inflammation of the endometrium was observed, characterized by an influx of neutrophils, macrophages and dendritic cells within 12 to 24 hours of mating (Hunt and Robertson, 1996; Robertson et al., 1996). Simultaneously it was demonstrated that semen induced cellular inflammation was due to increased expression of proinflammatory cytokines by the estrogen primed endometrial epithelium, including expression of *Csf2*, *Rantes*, *Mip1a* and *Mcp1* (Robertson and Seamark, 1992; Robertson et al., 1997). Furthermore, the active component of semen to elicit this response was identified as seminal fluid and not spermatozoa (Robertson et al., 1996), while additional studies showed seminal fluid transforming growth factor beta (TGFβ) as the bioactive molecule in semen of mice responsible for postcoital inflammation (Tremellen et al., 1998). Similar effects were characterized in the human cervix, where by semen exposure could induce cellular inflammation 24 hours after coitus, while seminal fluid or TGFβ would modulate the gene expression of proinflammatory cytokines in cultured cervical cells (Pandya and Cohen, 1985; Thompson et al., 1992; Sharkey et al., 2007; Sharkey et al., 2012a; Sharkey et al., 2012b). Exposure of cultured bovine endometrial epithelial cells, stromal cells or explants to seminal fluid or TGFβ increases the expression of *IL1B*, *TNF* and *IL6* expression, while intrauterine infusion of seminal fluid had very little effect on endometrial gene expression at 12 or 24 h post infusion (Ibrahim et al., 2019; Rizo et al., 2019). Interestingly, bovine seminal fluid has been shown to possess significant RNAse activity which could be determinantal to cell viability (Fernandez-Fuertes et al., 2019). Regardless of the mixed results from species, the physiological function of postcoital inflammation is still not clear.

Following allogeneic mating in the rodent the lymph nodes that drain the uterine tissues and the spleen transiently enlarge due to an influx of lymphocytes and antigen presenting cells that had phagocytosed spermatozoa (Beer and Billingham, 1974; Watson et al., 1983; Parr and Parr, 1990). When the phenotype of lymphocytes in uterine draining lymph nodes were characterized, it was revealed that exposure to semen 4 days prior, or specifically seminal fluid increased the development of regulatory T cells with tolerogenic function directed to paternal antigens found in semen (Robertson et al., 2009; Guerin et al., 2011; Robertson et al., 2013). The consequence of maternal tolerance to paternal antigen dates back to the postulates of Sir Peter Medawar who suggested that specific mechanisms of maternal tolerance must exist to allow the survival of the semi-allogeneic fetus to survive gestation (Medawar, 1953). More recently, it was shown in the mouse that maternal tolerance to paternal antigens via regulatory T cells is critical in the survival of the fetus (Aluvihare et al., 2004). It is plausible that maternal tolerance is initiated at the time of insemination when the maternal immune system is first exposed to paternal antigen and that postcoital inflammation is responsible for the trafficking of paternal antigen in the form of spermatozoa to the uterine draining lymph nodes and drive the expansion of T regulatory cells in preparation for embryo implantation. However, no or little evidence for these mechanisms exist in species other than the mouse.

Embryo transfer to unmated recipients (with no exposure to semen) routinely establishes healthy pregnancies in many species including rodents, humans, swine, cattle, horses etc indicating that female exposure to semen is not a necessity for pregnancy. Indeed, if seminal fluid is supplemented at the time of artificial insemination using semen that contains little to no seminal fluid, pregnancy rates are not increased in cattle or swine (Murray et al., 1983; Odhiambo et al., 2009; Ortiz et al., 2019). However, while seminal fluid supplementation at the time of insemination does not increase pregnancy rates in swine, evidence does suggest that additional seminal fluid exposure increases litter size and embryo development (Murray et al., 1983; O'Leary et al., 2004). Two large studies in cattle using over 3,500 cattle subjected to timed artificial insemination supplemented with seminal fluid or saline saw no effect on conception rate, pregnancy loss, calving rate or gestation length (Odhiambo et al., 2009; Ortiz et al., 2019). However, it was observed that primiparous dams bred with X-sorted semen and supplemented with seminal fluid at the time of insemination produced heifer calves that were heavier at birth compared to dams supplemented with saline (Ortiz et al., 2019). Separately, transfer of blastocysts to heifers seven days after mating to vasectomized bulls increased the length of recovered filamentous embryos and altered trophectoderm gene expression compared to heifers that were not mated (Mateo-Otero et al., 2020). Collectively, these findings suggest that seminal fluid exposure at the time of insemination may not be critical for pregnancy establishment but could alter pregnancy outcomes by increasing litter size or programing fetal growth. In mice, the surgical removal of the seminal vesicle glands that produce the vast majority of seminal fluid does decreases pregnancy rates by 65% and the number of implantations by 37% when sires are used for natural mating. Altered pregnancy rates in these mice is likely due to alterations in sperm transport and viability, as fertility can be restored if 2-cell or blastocyst stage embryos are transferred to pseudopregnant recipients mated with vasectomized males that have also had the seminal vesicles removed (Bromfield et al., 2014). Interestingly, offspring produced by seminal vesicle excised sires have increased placental weight, grow heavier in adulthood, have increased adiposity and altered metabolic activity, suggesting that the seminal vesicle gland secretions alter postnatal phenotype of offspring (Bromfield et al., 2014). The authors also demonstrate that the surgical removal of the seminal vesicle gland reduces early embryo development due to altered expression of embryotropic factors in the oviduct, which they conclude is partially responsible for the altered adult phenotype observed in offspring, concordant with the fetal programing hypothesis (Barker, 1998). Interestingly, the same increased adiposity is observed in male offspring produced by the transfer of 2-cell embryos into pseudopregnant recipients mated with vasectomized males that have also had the seminal vesicles removed, but not if blastocyst embryos are transferred, further providing evidence that the oviductal response to seminal fluid is altering pregnancy outcomes in the mouse. Conversely, bulls that have had the seminal vesicle glands surgically excised have reduced ejaculate volume but no reduction in conception rates when used for breeding (Faulkner et al., 1968).

While numerous studies have demonstrated specific alterations to maternal tissues in response to semen of various species, only three studies exist that show that maternal exposure to seminal fluid alters pregnancy outcomes – one in the mouse in which adult offspring are altered in the absence of seminal fluid, one in swine in which seminal fluid supplementation increases litter size, and one in cattle in which seminal fluid supplementation increased birth weight of heifer calves (Murray et al., 1983; Bromfield et al., 2014; Ortiz et al., 2019). However, it must be clarified that the intrauterine infusion of seminal fluid performed in cattle is a pharmacological application as it does not mirror the physiological deposition of semen into the vagina during live cover with a bull. In parallel, pregnancies are routinely initiated in the absence of seminal fluid in multiple species, but it is unclear if the outcomes of these pregnancies are optimal or could be improved if seminal fluid exposure of maternal tissues was to occur at the time of conception.

## What we think we know about the consequences of female tissue responses to semen

Achieving pregnancies in the absence of semen and postcoital inflammation is commonplace in many species including rodents, humans and many domestic species. In 2020, 203,164 human IVF cycles were initiated in the United States with the intent of embryo transfer, of which 165,041 transfers occurred, resulting in 91,453 pregnancies (55%) and 75,023 live birth pregnancies (45%) (National Center for Chronic Disease Prevention and Health Promotion, Division of Reproductive Health, 2020). According to data obtained by the International Embryo Technology Society, in 2022 a total of 1,189,699 embryos were transferred globally into cattle, 41,316 into sheep, 20,381 into goats, and 28,996 into horses (Viana, 2023). The numbers of embryo transfers for domestic species pales into insignificance compared to the number of bovine semen units sold by the United States for artificial insemination, with over 69 million units sold in 2022 (Weiker, 2023). The processing of semen for artificial insemination varies amongst companies, but all entail the significant dilution of semen in extenders that contain various compounds including antibiotics, lipids sourced from animal products like hen eggs or proprietary synthetic compounds, effectively reducing the volume of semen and seminal fluid encountered by the female reproductive tract (Lonergan, 2018). Additionally, sexed semen contains only spermatozoa diluted in artificial semen extender, effectively eliminating exposure of the female to seminal fluid. Collectively it is important to consider the sheer numbers of cattle born by embryo transfer or artificial insemination where postcoital inflammation is either absent or significantly reduced. And while conception rates are marginally increased in cows bred by natural service with a bull compared to artificial insemination, this is due to the increased opportunities for breeding when live cover is used in place of artificial insemination (Lima et al., 2009). The simple realization that millions of pregnancies have been achieved in cattle and humans by using embryo transfer whereby female tissues are never exposed to semen, one may ask, what is the potential physiological consequence of maternal responses to semen and the postcoital inflammatory response?

It has been proposed that maternal exposure to semen improves pregnancy outcomes and diminishes pregnancy complications by facilitating the improved development of the preimplantation embryo due to increased expression of embryotropic molecules in the female tract, modulating maternal immune tolerance to the semiallogeneic conceptus and altering tissue remodeling of the endometrium to allow for improved implantation and placentation (Robertson and Sharkey, 2001; Robertson, 2005; Bromfield, 2014, 2016, 2018). However, very little direct experimental evidence exists to support these theories. As described above, one study has demonstrated a reduced preimplantation embryo quality when seminal vesicle excised males are used to generate pregnancies in the mouse, and two studies (one in the pig and one in the bovine) have suggested improved embryo quality when females are exposed to seminal fluid – neither of which demonstrated altered pregnancy outcomes (O'Leary et al., 2004; Bromfield et al., 2014; Mateo-Otero et al., 2020). While semen does induce the expression of embryotropic factors such as CSF-2, IL-6 and LIF in the female reproductive tract of many species, in vitro culture of embryos demonstrates that these factors are not an absolute requirement to embryo development or pregnancy success (Schluns et al., 1997; Bromfield et al., 2014). However, the supplementation of embryo culture medium with CSF-2, IL-6 or LIF has been shown to improve embryo development and potentially even program offspring development after parturition in mice and cattle (Mitchell et al., 2002; Sjoblom et al., 2005; Block et al., 2011; Kannampuzha-Francis et al., 2015; Wooldridge and Ealy, 2019).

Maternal immune tolerance to the semiallogeneic conceptus during pregnancy is considered an absolute requirement for successful pregnancy outcomes (Medawar, 1953). The mechanisms by which maternal tolerance to paternal antigen is achieved has yet to be fully elucidated; however, exposure to semen at conception has been proposed as one mechanism in the rodent. The true nature for requirements of maternal immune tolerance are evident in the mouse, as depletion of peripheral CD25 positive T regulatory cells renders females infertile (Aluvihare et al., 2004). In parallel, the expansion of T regulatory cells in the mouse and pig oviduct, endometrium, and draining lymph nodes of the uterus is in part facilitated by exposure to seminal fluid at the time of mating (Robertson et al., 2009; Jiwakanon et al., 2010; Guerin et al., 2011). However, alternative mechanisms for the expansion of these important cell types must exist to allow for pregnancy establishment after embryo transfer.

Secondary associations of pregnancy success and semen exposure exist as part of epidemiological studies. In women receiving embryo transfer, it is routine to abstain from coitus around the time of transfer to avoid uterine contraction and infection that could terminate a pregnancy (Fanchin et al., 1998a; Fanchin et al., 1998b). However, a single study where patients were asked to engage in intercourse around the time of embryo transfer demonstrated a small yet significant increase in the proportion of women with a biochemical pregnancy compared to patients that abstained from intercourse (11.0% *vs.* 7.7%) (Tremellen et al., 2000). Similarly, the maternal response to semen has been suggested to reduce pregnancy complications including preeclampsia, recurrent miscarriage and fetal growth restriction. A positive association between the use of barrier contraception or short periods of cohabitation and preeclampsia has been established, whereby the authors suggest that increased exposure of maternal tissues to semen reduces the incidence of preeclampsia (Klonoff-Cohen et al., 1989; Robillard et al., 1994). Even more compelling is a study that utilized vaginal suppositories of seminal fluid to demonstrate a decrease in recurrent miscarriage (Coulam and Stern, 1995). While these studies suggest a positive association of semen exposure and pregnancy outcomes, to date no mechanism has been able to directly link pregnancy outcomes and the postcoital response to semen in humans or animals other than the mouse.

In the mouse, TGFβ derived from seminal fluid has been shown to be the most potent bioactive molecule to elicit the postcoital inflammatory response in maternal tissues and drive the expansion of T regulator cells (Tremellen et al., 1998; Robertson et al., 2002; Fantini et al., 2006; Konkel et al., 2017). Similarly, TGFβ is present at high concentrations in human, boar and bull semen and can partially mimic the actions of semen or seminal fluid in cultured cells (Nocera and Chu, 1995; Robertson et al., 2002; Sharkey et al., 2002, 2012a; O'Leary et al., 2011; Rizo et al., 2019). However, the supplementation of TGFβ at the time of artificial insemination in cattle does not improve pregnancy rates and no correlation between semen TGFβ and fertility exists in boars or humans (Loras et al., 1999; von Wolff et al., 2007; Odhiambo et al., 2009; O'Leary et al., 2011).

## What we need to do if we envisage using semen components to modulate pregnancy outcomes

Assisted reproductive technologies like embryo transfer and artificial insemination reduce or eliminate exposure of the maternal reproductive tract to semen and/or seminal fluid. The utility of advanced technologies including in vitro fertilization, embryo culture and intracytoplasmic sperm injection in the human have been associated with increased pregnancy failures, increased pregnancy complications and increased birth defects in the human (Cox et al., 2002; Hansen et al., 2002; Sutcliffe et al., 2002; Kurinczuk et al., 2004; Hansen et al., 2005; Ludwig et al., 2005). The vast majority of these studies conclude that the extreme manipulation of gametes during these procedures and suboptimal culture of embryos results in imprinting and genetic defects responsible for the increased complications observed in human patients. In cattle however, two unique studies exist to suggest that advanced reproductive technologies also diminish the genetic potential of offspring (Siqueira et al., 2017; Lafontaine et al., 2023). These studies utilized the genomic potential of dairy cows and compared it to the actual phenotypic outputs of milk production in a total of 319,140 animals conceived by artificial insemination using conventional semen or X-sorted semen, multiple ovulation embryo transfer or in vitro fertilization with embryo transfer. Collectively these studies suggest that as the complexity of technology for conception increased the cumulative mortality of offspring increased and their productive phenotype did not reach their genomic potential (Siqueira et al., 2017; Lafontaine et al., 2023). The question from these impressive studies remains, what is the mechanisms by which the phenotype of these offspring is altered following assisted reproductive technologies, and could semen exposure play a role?

While we have discussed three experiments (one each in mouse, cattle and swine) that demonstrate a definitive link between improved pregnancy outcomes and semen exposure at the time of conception, many unanswered questions pertaining to the relevance of physiological semen exposure and pregnancy outcomes remain. Various experimental paradigms have shown the influence of semen, seminal fluid or bioactive semen components on maternal responses that vary from postcoital cellular inflammation, altered endometrial or oviductal gene expression, modulation of lymphocyte populations and even embryo quality in different species. However, key questions remain that would allow us to draw conclusive, mechanistic effects of how semen exposure modulates pregnancy outcomes. The first and most important question pertains to physiological semen exposure; while many studies focus on the rodent where the uterus is exposed to large volumes of semen, fewer studies have focused on the effects of vaginal semen exposure on promoting pregnancy outcomes in species like the human or cattle. The singular study in cattle that demonstrated an effect of seminal fluid exposure on birth weight utilized intrauterine infusion of pooled seminal fluid. It is unclear if any seminal fluid reaches the upper reproductive tract in cattle (or humans), and as such this experiment was indeed a pharmacological intervention. Indeed, when heifers were mated with intact bulls a total of 22 differentially expressed genes were identified in the endometrium 24 h later when compared to heifers that were not mated; whereas no differentially expressed genes were identified in the endometrium when heifers were mated with vasectomized bulls (compared to heifers that were not mated), suggesting that vaginal exposure to semen or seminal fluid by copulation has very little effect on the endometrium (Recuero et al., 2020). It will be imperative to determine in all species how much, if any, seminal fluid reaches the upper reproductive tract if we hope to understand the physiological significance of semen exposure. Similarly, only a single study in each of the pig, cow and mouse have shown that semen exposure alters development of the preimplantation embryo. It is unclear how the increased birth weight of calves is driven by seminal fluid exposure, is this due to altered embryo development, interaction of spermatozoa with seminal fluid, altered placentation, and are there further consequences to the offspring that are born slightly heavier than their herd mates? Similarly, do semen induced changes to peripheral immune cell populations have any effect on pregnancy success or are these simply alterations driven by semen exposure that do not have a specific physiological function associated with pregnancy outcome, and do these alterations exist in animals other than mice? Beyond the physiological responses to semen, if the intent is to develop pharmacological interventions or semen derived supplements to improve pregnancy outcomes it will be important to identify the bioactive compounds of semen that elicit the maternal responses involved in modulating pregnancy outcomes. Semen is a complex biological fluid, and its composition varies dramatically from one species to another, within an individual over time and is susceptible to exogenous influence by diet and environment. It is likely that all of these types of questions are pertinent to understanding the true physiological role of semen exposure in the female, more over it will be important to understand these in relevant species in which we are trying to improve pregnancy outcomes like the human or domestic species and less so in the mouse.

## Conclusions

There is indisputable evidence that semen interacts with maternal tissues at the time of insemination to elicit distinctive cellular and molecular alterations to reproductive and peripheral tissues. The consequence of semen exposure on pregnancy outcomes is less established, especially beyond the mouse, with minimal evidence that seminal fluid supplementation increases litter size in swine and heifer birth weight in cattle. While these studies are of great interest to animal agriculture, they do not directly speak to physiological responses due to the use of artificial insemination with diluted semen and intrauterine application of additional seminal fluid that would normally be found only in the vagina. While I and others will continue to explore how semen interacts with maternal tissues, we have a long journey ahead to determine a physiological role and potentially develop therapeutics to improve pregnancy outcomes in domestic species and humans.
